# Hypermethylation of genomic 3.3-kb repeats is frequent event in HPV-positive cervical cancer

**DOI:** 10.1186/1755-8794-2-30

**Published:** 2009-05-27

**Authors:** Alexey N Katargin, Larissa S Pavlova, Fjodor L Kisseljov, Natalia P Kisseljova

**Affiliations:** 1 Laboratory of Molecular Biology of Viruses, Institute of Carcinogenesis, NN Blokhin Cancer Research Center, Russian Academy of Medical Sciences, Moscow, Russia

## Abstract

**Background:**

Large-scale screening methods are widely used to reveal cancer-specific DNA methylation markers. We previously identified non-satellite 3.3-kb repeats associated with facioscapulohumeral muscular dystrophy (FSHD) as hypermethylated in cervical cancer in genome-wide screening. To determine whether hypermethylation of 3.3-kb repeats is a tumor-specific event and to evaluate frequency of this event in tumors, we investigated the 3.3-kb repeat methylation status in human papilloma virus (HPV)-positive cervical tumors, cancer cell lines, and normal cervical tissues. Open reading frames encoding DUX family proteins are contained within some 3.3-kb repeat units. The *DUX *mRNA expression profile was also studied in these tissues.

**Methods:**

The methylation status of 3.3-kb repeats was evaluated by Southern blot hybridization and bisulfite genomic sequencing. The expression of *DUX *mRNA was analyzed by RT-PCR and specificity of PCR products was confirmed by sequencing analysis.

**Results:**

Hypermethylation of 3.3-kb repeats relative to normal tissues was revealed for the first time in more than 50% (18/34) of cervical tumors and in 4 HPV-positive cervical cancer cell lines. Hypermethylation of 3.3-kb repeats was observed in tumors concurrently with or independently of hypomethylation of classical satellite 2 sequences (Sat2) that were hypomethylated in 75% (15/20) of cervical tumors. We have revealed the presence of transcripts highly homologous to *DUX4 *and *DUX10 *genes in normal tissues and down-regulation of transcripts in 68% of tumors with and without 3.3-kb repeats hypermethylation.

**Conclusion:**

Our results demonstrate that hypermethylation rather than hypomethylation of 3.3-kb repeats is the predominant event in HPV-associated cervical cancer and provide new insight into the epigenetic changes of repetitive DNA elements in carcinogenesis.

## Background

DNA methylation plays an important role in the maintenance of the gene expression program and genome stability. Tumor cells are characterized by a paradoxical change in DNA methylation pattern: global DNA demethylation and local hypo- and hypermethylation of gene regulatory regions that result in transcriptional activation of oncogenes and inactivation of tumor suppressors. Current opinion is that the targets for aberrant demethylation in tumors are represented by DNA sequences that are methylated in normal tissues. Repetitive elements are considered among such DNA sequences. Frequent hypomethylation of repetitive elements is observed in diverse human cancers and is thought to largely account for genomic instability [1-3].

Cervical carcinomas are the second most frequent type of cancer in women. Infection with high risk type human papilloma viruses (HR-HPV) is the main risk factor for cervical cancer [4]. HR-HPV infections are extremely widespread throughout the world, but malignant transformation is a rare consequence of the infection. The progression from precursor intraepithelial lesions to cervical carcinomas is accompanied by additional genetic and epigenetic changes that have not been fully characterized. A low overall m5C content compared to normal cervical tissue was demonstrated in cervical cancer, although the methylation status has not been studied in any repetitive elements except LINE-1 [5,6].

We previously conducted a genome-wide differential analysis of DNA methylation in cervical cancer and normal tissues using methylation-sensitive arbitrarily primed PCR [7] and identified CpG islands located within 3.3-kb repeats as hypermethylated in tumors.

Members of the 3.3-kb repeat family reside in the subtelomeric regions of chromosomes 4 and 10 as tandemly arranged polymorphic arrays containing from 11 to 100 monomers. In addition, they are present on the short arms of all acrocentric chromosomes as interspersed repeats [8-11]. The subtelomeric region 4q35 named D4Z4 receives more attention because of its association with facioscapulohumeral muscular dystrophy (FSHD), an autosomal dominant disorder linked to partial deletion of the D4Z4 repeat array. At least one sequenced copy of D4Z4 contains a single-exon open reading frame (ORF) and an upstream region that can function as a promoter in reporter assays [12]. The ORF encodes a polypeptide called DUX4 that has a high level of similarity to the paired-type homeodomain proteins of families Pax and Otx [13]. *DUX4 *and *DUX4*-related transcripts (*DUX1*, -3, -4c -5, and -10) have been revealed in some human cells [14-18]. DUX1 and DUX4 have the properties of a transcription factor, and the products of exogenous translation of mRNAs of these genes localize to the cell nucleus [12,15,19].

In many normal somatic tissues 3.3-kb repeats are mostly but not completely methylated, and they are hypomethylated in glioblastomas, leukemia cell lines, tissues of patients with FSHD and with DNA methyltransferase deficiency syndrome ICF [20-24]. It has recently been shown that 3.3-kb repeats in the D4Z4 array are hypomethylated in some ovarian and Wilms tumors and are hypermethylated in other tumors of the same types relative to normal tissues [25].

To determine whether hypermethylation of 3.3-kb repeats is an event specific for HPV-positive cervical tumors and to evaluate the frequency of this event, we examined the methylation status of 3.3-kb repeats in tumors, cervical cancer cell lines, and normal tissues. We have demonstrated for the first time that more than 50% of cervical cancers display hypermethylation of 3.3-kb repeats compared to normal cervical tissues. Different regions within the 3.3-kb monomer exhibit inter-individual variation in the methylation patterns in normal tissues and different susceptibility to hypermethylation in tumors. We have shown that the 3.3-kb repeat hypermethylation can occur in tumors concurrently with hypomethylation of classical satellite 2 (Sat2) sequences that, according to many studies, are targets for demethylation in different tumor types [1-3]. We also revealed the presence of transcripts highly homologous to the *DUX4 *and *10 *genes in normal uterine cervical tissues and down-regulation of *DUX*-related transcripts in tumors with and without the 3.3-kb repeat hypermethylation.

## Methods

### Tissue samples

Squamous cell carcinomas and adenocarcinomas of uterine cervix and samples of adjacent normal tissues were obtained from patients with FIGO stages I, II, and III of the disease at the Blokhin Cancer Research Center. All tumors were HPV16/18-positive, and E7 viral genes were expressed in all of them according to PCR analysis [26]. Normal tissues of uterine cervix were obtained from patients with uterine polyps or tumors. All tissues were collected under the approval of the Institutional Review Board of the Blokhin Cancer Research Center. Informed consent was obtained from all patients. The study was done in accordance with the principles outlined in the Declaration of Helsinki.

### Cell lines

The HPV-positive human cervical carcinoma cells HeLa, SiHa, CaSki, and C4-I and the HPV-negative C-33A cell line (American Type Culture Collection, USA) were maintained in DMEM supplemented with 10% FCS. Cells were seeded at standard densities and treated with DNA methyltransferase inhibitor 5-aza-2'-deoxycytidine (5-aza-dC; Sigma, USA) for 3–4 days. The medium containing 5 μM 5-aza-dC was changed daily.

### DNA and RNA extraction

DNAs and RNAs were isolated from frozen tissues or cells by homogenization in 4.5 M guanidine isothiocyanate and centrifugation through a cesium chloride cushion as described previously [27].

### Bisulfite-based cytosine methylation analysis

The bisulfite conversion reaction was carried out using an EZ DNA Methylation Kit (Zymo Research, USA) according to the manufacturer's instructions under the following conditions: 15 cycles (30 s at 95°C and 15 min at 50°C). The DNA samples were used for the PCR amplification by JumpStart RedTaq DNA polymerase according to the manufacturer's instructions (Sigma, USA). Three sets of 3.3-kb repeat-specific primers corresponding to three regions on the upper strand of bisulfite-modified DNA were used: (1) Sq-1, sense GAA GGT AGG GAG GAA AAG (pos. 566) and antisense ACT CAA CCT AAA AAT ATA CAA TCT (pos. 796); (2) Sq-2, sense TAT GAA GGG GTG GAG TTT G (pos. 1619) and antisense AAA TAC CAA TAA CCT AAA CCA AC (pos. 1955); (3) Sq-3, sense AGT TTG GAG TTT TTG TAGTAG G (pos. 2942) and antisense CAA AAA TCC CAA ACC AAT CAA CC (pos. 3192) [23]. Hereafter the positions of primers are given according to the 3.3-kb monomer sequence [GenBank: AF 117 653]. The PCR products were cloned into pGEM-T Easy vector (Promega, USA) and used for automated sequencing (ABI Prism 3100-Avant Genetic Analyzer, Applied Biosystems, USA).

### Southern blot analysis

DNA samples (10 μg) were digested overnight with methylation-sensitive restriction endonucleases (Fermentas, Lithuania), separated in 1% agarose gel, transferred to a nylon membrane (Zeta-Probe, Bio-Rad, USA), and hybridized with [^32^P]-labeled PCR DNA probes for 3.3-kb repeat or Sat2-specific oligonucleotide TCG AGT CCA TTC GAT GAT [28]. Hybridization was performed in 7% (w/v) SDS (Sigma), 0.5 M sodium phosphate (pH 7.2), and 10 mM EDTA at 65°C for the PCR probes or at 42°C for the Sat2 oligonucleotide. Some membranes were rehybridized with the Sat2 oligonucleotide in 50% formamide at 42°C. The membranes were washed 2 or 3 times in 2× SSPE/0.1% SDS and in 0.1× SSPE/1% SDS at 55 and 42°C for the PCR and Sat2 probes, respectively, and exposed to X-ray film at -70°C. Intensities of hybridizing signals were determined for paired normal and tumor samples or for each cell line and three samples of healthy cervical tissues from patients with non-cervical pathology in the same blot using the Image-J 1.36b NIH software. The approximate extent of hyper- or hypomethylation was calculated in that way: the ratio of the high-molecular-weight hybridizing signal (>4.4 kb) to the total hybridization signal for each tumor (T) was divided by the analogous ratio for matching normal tissue (N). To calculate T/N for cell lines, the average from N for three healthy cervical tissue samples was used.

### Reverse transcription PCR

RT-PCR was carried with total cellular RNA treated with DNase I (Invitrogen, USA) using reverse transcriptase (Superscript II RNase H^-^, Invitrogen) according to the manufacturer's instructions. DNA contamination was controlled by conducting the same reaction without reverse transcriptase for each RNA sample. PCR was performed with the *DUX4*-specific primers (sense CCA CGG AGA CTC GTT TGG AC, pos. 1863, and antisense CCT GGA AAG CGA TCC TTC TCA, pos. 2141) under the following conditions: 94°C for 5 min; 35 cycles (94°C, 30 s; 64°C, 30 s; 72°C, 40 s), and at 72°C for 5 min. To control the content of the initial total mRNA, PCRs were carried out with the primers for *GAPDH *(sense ACC ACA GTC CAT GCC ATC AC, antisense TCC ACC ACC CTG TTG CTG TA) under the following conditions: 94°C, 5 min, 26 cycles (94°C, 30s; 58°C, 30s; 72°C, 1 min,) and 72°C for 5 min. After agarose gel electrophoresis, the PCR products were visualized under ultraviolet light or transferred to a membrane (Hybond-N+, Amersham Biosciences) and probed with *DUX4*-specific [^32^P]-labeled internal oligonucleotide TTG GTT TCA GAA TGA GAG GTC ACG (pos. 1996). Intensities of all bands were determined using the Image-J 1.36b NIH software. DUX-specific signals were normalized to GAPDH-specific signals in each sample, and the ratios N/T were calculated. The average from five healthy cervical tissue samples was used for unpaired tumor samples. Threefold change in tumors relative to normal tissues was taken into account.

### Statistical analysis

Nonparametric Wilcoxon matched pairs test was used to evaluate the methylation level differences between tumors and adjacent normal tissues. Fisher's exact test (two tailed) and χ-square test were used to compare frequencies of hypermethylation in tumors and normal tissues and portions of methylated CpG sites in different 3.3-kb monomer regions, respectively. Differences were considered statistically significant when P-values were below 0.05. All statistical procedures were performed using STATISTICA 6 software (StatSoft).

## Results

### Methylation pattern of 3.3-kb repeats in normal cervical tissues by bisulfite genomic sequencing

**Figure 1:**
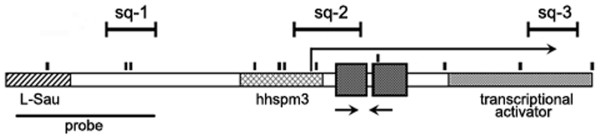
**Diagram of a 3.3-kb monomer**. Two squares denote double homeobox region. Sq-1, Sq-2, and Sq-3 mark the regions selected for sequencing analysis. The hybridization probes and RT-PCR primers are indicated by horizontal lines and arrows, respectively. The broken arrow indicates the position of *DUX4 *transcription start site. Vertical lines indicate *NaeI *sites.

**Figure 2:**
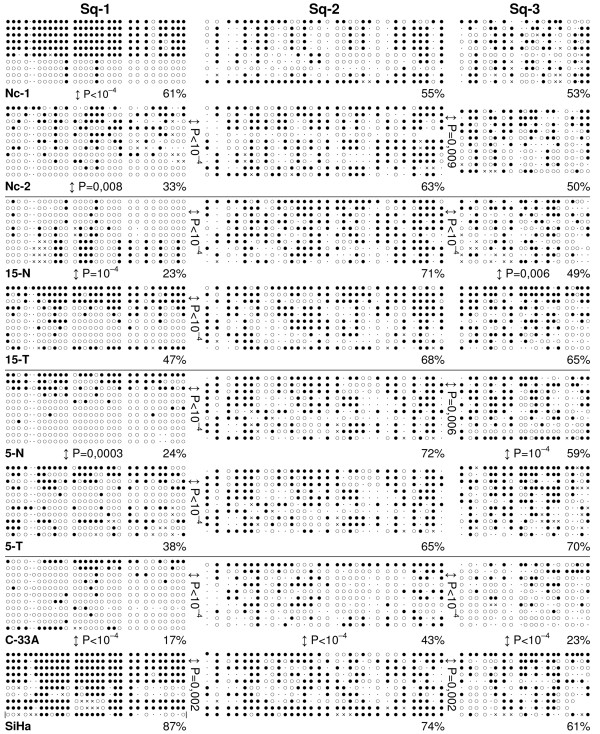
**Methylation status of 3.3-kb repeats by sodium bisulfite sequencing in cervical tissues, tumors, and cell lines**. Sq-1, Sq-2, and Sq-3 mark three regions of 3.3-kb monomer selected for sequencing. Each row of circles represents a cloned DNA molecule; closed circle, methylated CpG; open circle, unmethylated CpG; dot, CpG site lost from the consensus sequence due to mutations; cross, CpG site status was not determined. The percentage of methylated CpGs among all CpGs analyzed is given below each sample. Nc, normal cervix from non-cervical-cancer patients; T, tumor; N, tumor – tumor-adjacent normal tissue. Numbers of patients are indicated as in Figure 3C. Only P values below 0.05 are given (χ-square test).

First, the 3.3-kb repeat methylation pattern was evaluated in normal cervical tissues, which has not been reported previously. Three regions within the 3.3-kb monomer were selected for bisulfite genomic sequencing (Figure 1): the 5' region (Sq-1); the middle region (Sq-2) around the *DUX-4 *transcriptional start site that was described for a single copy cloned from the 4q35 D4Z4 array of an FSHD patient [12]; and the 3' region (Sq-3), which was shown to be hypomethylated in glioblastomas [24]. Bisulfite sequencing analysis can detect all closely homologous 3.3-kb copies from D4Z4 and other loci of the genome. Normal cervical tissues from two non-cervical cancer patients (Nc-1 and Nc-2) and two normal tissues adjacent to cervical tumors (15-N and 5-N) were used (Figure 2). The examined regions were unequally methylated in all normal cervical tissues. The levels of methylation varied from 23–61% for Sq-1 to 55–72% for Sq-2 and 49–59% for Sq-3. In three normal tissues, the Sq-1 regions were significantly less methylated compared to the Sq-2 or Sq-3 regions (P ≤ 10^-4^). In addition, significant interindividual variations in the 3.3-kb repeat methylation patterns were revealed. Normal tissues could differ from one another in the local methylation level (Nc-1, Nc-2, 5-N and 15-N, Sq-1 regions, P ≤ 0.008; Nc-1 from 5-N and 15-N, Sq-2 regions, P < 10^-3^). Finally, these results demonstrated that normal cervical tissues exhibited a considerable but incomplete methylation of 3.3-kb repeats just as shown previously for the 4q35 D4Z4 locus in other normal tissues by Southern blot analysis [21].

### Methylation status of 3.3-kb repeats in cervical tumors and cancer cell lines by bisulfite genomic sequencing

The changes in the 3.3-kb repeat methylation status in tumors were evaluated relative to paired normal tissues taking into account considerable interindividual variation in the repeat methylation pattern. Sequencing analysis demonstrated hypermethylation in two tumors relative to adjacent normal tissues (Figure 2, 5-T and 15-T). It is of interest that the levels of hypermethylation differed among the three regions. The methylation levels of the middle regions Sq-2 did not change substantially in either tumor compared to adjacent tissues (P > 0.05). The Sq-3 regions exhibited moderate but statistically significant levels of hypermethylation in both tumors (P ≤ 0.006). At the same time, a more strong increase in the Sq-1 region methylation levels (1.6 and 2 times) was seen in two tumors compared to matched normal tissues (P ≤ 3 × 10^-4^). The strongest level of hypermethylation (87%) was revealed in the Sq-1 region in HPV-positive cervical cancer cell line SiHa. Surprisingly, all three regions exhibited hypomethylation in HPV-negative cervical cancer cell line C-33A compared to all other samples examined. The local susceptibility to demethylation also varied (17%, 43%, and 23% of methylated CpG sites for Sq-1, Sq-2, and Sq-3 regions, respectively). These findings demonstrate that the Sq-1 region is more susceptible to changes in the methylation status than other regions and suggest that the changes in methylation status of 3.3-kb repeats in cancer cells may be sequence-specific.

### Frequency of hypermethylation of 3.3-kb repeats in cervical HPV-positive carcinomas

**Figure 3:**
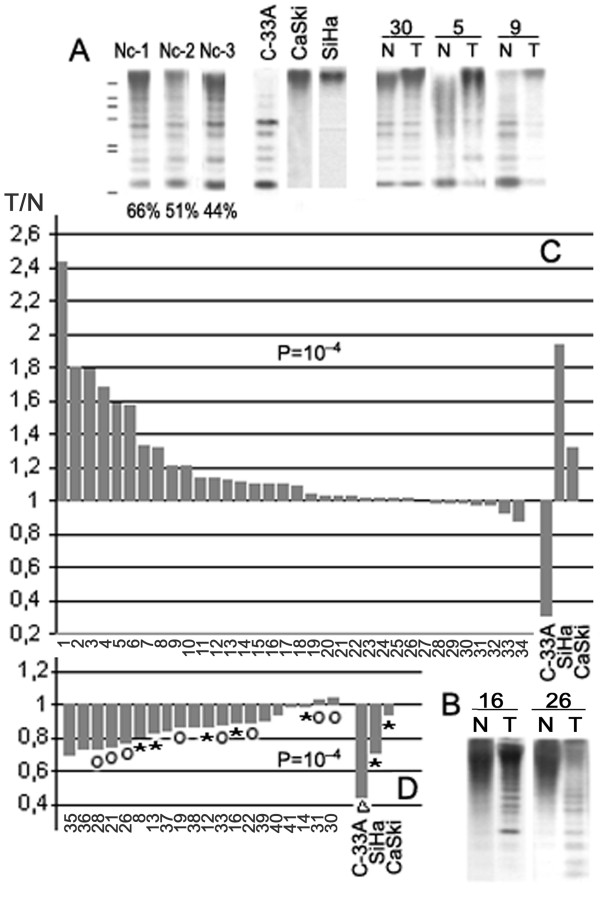
**Analisis of methylation status of 3.3-kb and Sat2 repeats in HPV-positive cervical tumors by Southern blot hybridization**. Nc, normal cervix from non-cervical-cancer patients; T, tumor; N, tumor-adjacent normal tissue. Lines to the left of the panel are molecular mass markers (from top to bottom, 24.3, 9.4, 6.6, 4.4, 2.3, 2.0, and 0.5 kb). **(A) **Hybridization with the 3.3-kb repeat-specific probe, *NaeI *digests of DNAs. Numbers under the panels indicate the percentage of the high-molecular-weight hybridization signal (>4.4 kb) to the total hybridization signal for each track. **(B) **Hybridization with the Sat2-specfic oligonucleotide, *Bst*BI digests of DNAs. **(C) **Hypermethylation of 3.3-kb repeats in tumors. **(D) **Hypomethylation of Sat2 in tumors. The asterisk and triangle indicate samples with hypermethylation and hypomethylation of 3.3-kb repeats, respectively; the circle – unchanged methylation status of 3.3-kb repeats.

The frequency of hyper- or hypomethylation of 3.3-kb repeats in cervical HPV-positive carcinomas was studied by Southern blot analysis of 34 paired samples using the fragment of the 5' end of the 3.3-kb monomer as a hybridization probe and *NaeI* methylation-sensitive restriction endonuclease (Figure 3). Eleven *NaeI* sites reside within the 3.3-kb monomer including two sites located within the Sq-1 region (Figure 1). This analysis shows the overall methylation status of all copies of the repeats. The methylation levels in tumors were compared to those in nonmalignant cervical tissues of the same patient taking into account individual variations of methylation patterns of 3.3-kb repeats. Indeed, the percentage of undigested DNA (high-molecular-weight fragments > 4.4 kb) of normal tissues from different individuals substantially varied, confirming interindividual distinctions in the 3.3-kb repeat methylation level revealed by sequencing analysis (Figure 3A, 44 to 66% in the same blot). Hypermethylation of 3.3-kb repeats was revealed in 18 out of 34 (53%) carcinomas compared to adjacent normal tissues (Figure 3C; P = 10^-4^). The samples with low (15-T) and middle (5-T) levels of hypermethylation determined by Southern blot exhibited clearly detectible hypermethylation status by bisulfite genomic sequencing (Figure 2). The hypermethylation of 3.3-kb repeats was shown also in HPV-positive line SiHa by both Southern blot and sequencing analysis, and both techniques demonstrated the 3.3-kb repeat hypomethylation in HPV-negative cell line C-33A. Thus, the results of both techniques coincide with each other and sequencing data verify the sensitivity of Southern blot for detecting differences in DNA methylation. None of the 34 HPV-positive tumors displayed such low methylation level as HPV-negative cell line C-33A did (Figure 3A and 3C). No correlation was found between the 3.3-kb repeat methylation status and clinicopathological data (age, tumor stage, and histological grade and type). This indicates that the 3.3-kb repeat hypermethylation may be an early event during cervical carcinogenesis. Thus, these results demonstrate that hypermethylation rather than hypomethylation of 3.3-kb repeats is a frequent event in HPV-positive cervical carcinomas.

### Both hypermethylation of 3.3-kb repeats and hypomethylation of satellite repeat Sat2 in cervical tumors and cancer cell lines

The high frequency of hypermethylation of 3.3-kb repeats in cervical cancer has not been reported earlier. In order to determine whether 3.3-kb repeat hypermethylation takes place in HPV-positive cervical tumors along with the well-known hypomethylation of different repeats in tumors, the methylation status of Sat2 was analyzed by Southern blot hybridization (Figure 3B and 3D). The Sat2 methylation status in normal and tumor cervical tissues was not known. Here, no low-molecular-weight fragments (< 4.4 kb) were revealed by Southern blot analysis in normal cervical tissues adjacent to tumors. This indicates that Sat2 is highly methylated in normal cervix just as in other human normal tissues. On the contrary, a large portion of the hybridization signal was observed in the low-molecular-weight fragments (< 4.4 kb) in tumors demonstrating hypomethylation of Sat2. Sat2 hypomethylation was revealed in 15 out of 20 (75%) tumors relative to normal adjacent tissues and in 5 cancer cell lines examined (Figure 3D; P = 10^-4^). Hypermethylation of 3.3-kb repeats and hypomethylation of Sat2 can coexist in the same tumor (Figure 3D and Additional file 1). On the other hand, Sat2 hypomethylation was observed in the tumors in the absence of 3.3-kb repeat hypermethylation indicating that the changes in the methylation pattern of different repeats are independent.

### Expression of DUX genes by RT-PCR

**Figure 4:**
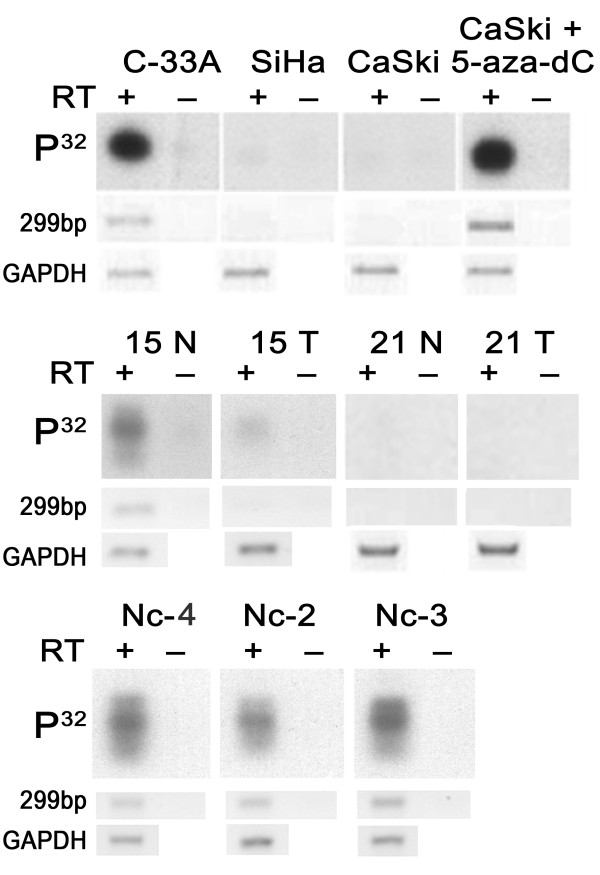
**Analysis of *DUX* gene expression in cervical tumors and cell lines by RT-PCR**. Top row: Southern blot hybridization of PCR products with the *DUX4*-specific [32] P-labeled oligonucleotide. Middle and bottom rows, electrophoregrams after ethidium bromide staining. *GAPDH*, a control for RNA stability and concentration. RT, reverse transcriptase; Nc, normal cervix from non-cervical-cancer patients; T, tumor; N, tumor-adjacent normal tissue. Patient numbers are as in figure 3.

The distribution of some *DUX*-related transcripts in normal and tumor cervical tissues was analyzed by RT-PCR with *DUX4*-specific primers. The products of RT-PCR of predicted size were revealed in normal uterine cervix and in normal tissues adjacent to cervical tumors. (Figure 4, Table 1). The expression levels in normal tissues were low compared to that of a control gene, GAPDH. To reveal the transcription suppression in tumors compared to normal tissues, RT-PCR products were analyzed by Southern blot hybridization with [^32^P]-labeled *DUX4*-specific oligonucleotide. In 30 out of 44 (68.2%) cervical carcinomas the [^32^P] RT-PCR bands were undetectable or faint compared to normal tissues. Twenty four tumors had matched normal tissues and 20 tumors were compared to the average from 5 healthy cervical tissue samples. The PCR products were absent in 5 out of 24 (20.8%) normal tissues adjacent to cervical tumors with suppression of *DUX *transcription. This indicates that interruption of *DUX *transcription may be an early event in carcinogenesis. The PCR products were also absent in 4 HPV-positive cancer cell lines.

**Table 1:** Frequency of down-regulation of expression of DUX-related genes in normal tissues and cervical carcinomas

Samples	No. tested	Down-regulation of mRNA
Normal cervical tissues	5	0

Adjacent to tumors nonmalignant cervical tissues	24	5 (20.8%)*
Tumors	44	30 (68.2%)
		P = 0.003**
		
Cervical cancer cell lines	5	4

Down-regulation was defined as the ratio normal/tumor exhibiting at least threefold reduction of mRNA level in tumor or the absence of mRNA expression.

* Products of PCR were absent in adjacent normal tissue as well as matched tumor.

** Fisher's exact test

The specificity of PCR amplification was tested by cloning the 299-bp RT-PCR products of C-33A cell lines and sequencing of 11 such clones. All copies were highly homologous to *DUX4*, *DUX4c *(100–94%), and *DUX10 *(99.7–89%). The cloned sequences were less similar to *DUX1-3 *and *DUX5 *(84–90%). One third of the transcripts had perfect ORFs. Thus, the transcripts revealed by RT-PCR in cervical cells are highly homologous to *DUX *family genes.

### Comparison of 3.3-kb repeat methylation status with DUX gene expression

The results of both 3.3-kb repeat methylation status and *DUX *gene expression were available for 24 carcinomas and matched normal tissue (Table 2). *DUX *mRNA expression was suppressed in 10 carcinomas with hypermethylation. Four highly methylated cell lines had no *DUX *transcripts. The transcripts were revealed in only one cancer cell line with hypomethylated 3.3-kb repeats (C-33A). Thus, there is a significant correlation between hypermethylation of 3.3-kb repeats and down-regulation of *DUX *transcription. This indicates that methylation plays a role in inactivation of *DUX *transcription. However, there were 8 out of 18 (44%) tumors that had lost *DUX *transcripts without change in methylation status compared to nonmalignant tissues. These findings suggest that there are other mechanisms besides DNA methylation underlying the suppression of *DUX *expression.

**Table 2:** Correlation between 3.3-kb repeats hypermethylation and loss of expression of DUX mRNA in cervical carcinomas and cancer cell lines

mRNA expression		hypermethylation	
		+	-
	Cervical carcinomas		
-	18	10	8
+	6	0	6
	Cell lines		
-	4	4	0
+	1	0	1

### Re-expression of DUX genes after treatment with 5-aza-dC

Re-expression of *DUX *genes after 5-aza-dC treatment was revealed in one out of four highly methylated cervical cell lines, CaSki, which confirms both the role of methylation in the expression control of *DUX *genes and the existence of other mechanisms of *DUX *gene silencing (Figure 4).

## Discussion

### Prevalence of hypermethylation over hypomethylation of 3.3-kb repeats in HPV-positive tumors

Demethylation at repeated DNA sequences is a common hallmark of human cancers and can be used as a surrogate marker of genome-wide methylation changes. [1-3]. We have demonstrated that the 3.3-kb repetitive elements are heavily but not completely methylated in normal cervical tissues and subject to hypermethylation in more than 50% of HPV-positive cervical tumors and four cervical cancer cell lines. It has been shown that 3.3-kb repeats are hypomethylated in glioblastomas and leukemia cell lines; they are hypomethylated in some portion of ovarian and Wilms tumors but hypermethylated in another portion of these tumors [23-25]. We observed strong hypomethylation of 3.3-kb repeats relative to normal cervical epithelium only in HPV-negative cancer cell line and have concluded that 3.3-kb repeat hypermethylation is the predominant event in cervical cancer. There were no HPV-negative cervical tumors among our samples, and the methylation status of 3.3-kb repeats in them remains to be evaluated, but one cannot exclude that the prevalence of the 3.3-kb repeat hypermethylation in cervical tumors is related to HPV genome persistence. HR-HPVs induce carcinomas by expressing viral oncogenes E6 and E7 [4]. E7 deregulates several cellular pathways. In particular, it inactivates the retinoblastoma tumor suppressor protein (Rb) [29]. In normal cells, Rb regulates DNA methyltransferase 1 (DNMT1) that maintains DNA methylation during S phase of the cell cycle by at least two mechanisms. Rb directly interacts with and modulates DNMT1 activity, and Rb is required for proper cell cycle regulation of Dnmt-1 transcription [30,31]. Oncogene E7 disrupts this regulation, abolishing Rb; in addition, it directly interacts with and activates DNMT1 [32]. It has been shown recently that DNMT1 is necessary to maintain the 3.3-kb repeat methylation [33]. The fine mechanisms responsible for changes of 3.3-kb repeat methylation status remain to be clarified, but the persistence of E7 in HPV-positive tumors likely ensures prevalence of hypermethylation over hypomethylation of 3.3-kb repeats in contrast to virus-negative tumors where hypomethylation or both processes may take place. Other tandem repeats, NBL2, have been found to be hypomethylated in some types of cancer but hypermethylated in others [34,35]. Taken together, these data bring up the question of tumor-type specificity in epigenetic changes of repeats.

### Sequence-specific 3.3-kb repeat methylation

We have demonstrated that different regions of the 3.3-kb monomer were irregularly methylated in normal tissues and were subject to hypermethylation independently from one another in the same tumor. The 5' region (Sc-1) was less methylated in normal tissues but more susceptible to both hypomethylation and hypermethylation in tumor cells than other regions. Recently, the presence of a hypermethylation-resistant region within the 3.3-kb monomer was shown in ovarian and Wilms tumors [25]. This hypermethylation-resistant region is located only 70 and 100 nucleotides upstream from our Sq-2 region and the TATA box, respectively. The Sq-2 region is noticeably methylated in normal tissues, and its methylation level showed no significant changes in tumors. Overall, at least three subregions with different status and susceptibility to hypermethylation have been found within the 5' half of the 3.3-kb monomer [25] and the Sq1 and Sq2 regions. Both hypomethylation and hypermethylation of different CpG dinucleotides was shown in a short region of NBL2, a tandem DNA repeat, in tumors [36].

Hence, these studies and our data demonstrate that changes in the methylation status of genomic repeats in tumors may be sequence-specific. This indicates that the choice of the region to test may greatly influence conclusions reached by studying the methylation status of DNA repeats in cancer.

Different local susceptibility to methylation can be due to the complex structure of the 3.3-kb monomer, which includes several known sequence motifs: double homeobox, LSau, and hhspm3 [8,37]. Several regulator sequences have been found within these motifs. The 5' region of the 3.3-kb element studied here (Sq-1) is about 200 bp downstream from the LSau motif (Figure 1). LSau contains an exceptionally strong transcriptional enhancer [38]. Our middle region of the 3.3-kb element (Sq-2) includes a fragment of hhspm3. This region can function as a promoter in reporter assays and includes a TATA box and a transcription initiation site of *DUX4 *[12,16]. It has been shown that the multiprotein complex containing the transcription activator/repressor YY1 binds this region in HeLa cells and represses transcription of genes located upstream from D4Z4 [39]. The 3' end of the 3.3-kb element was shown to have a strong activator in a reporter assay [40]. Our hypermethylation-susceptible Sq-3 region lies within this fragment. It is possible that the interplay between the system of DNA methylation and regulatory factors binding these different regions determines their methylation levels in normal tissues and their susceptibility to hypermethylation in tumors. It is also possible that tissue-specific regulatory factors determine tumor-type specificity in epigenetic changes of 3.3-kb repeats discussed above.

### Both Sat2 hypomethylation and 3.3-kb repeat hypermethylation in tumors

We observed hypomethylation of Sat2 in cervical tumors along with hypermethylation of 3.3-kb repeats. It has been shown that Sat2 hypomethylation is significantly associated with global DNA hypomethylation in different tumors [2,3]. Thus, hypermethylation of 3.3-kb repeats does not interfere with the process of DNA hypomethylation in tumors. The mechanisms underlying these opposite processes are not known. Both Sat2 and 3.3-kb repeats are targets of DNMT3b, but only the latter is a target of DNMT1 [28,33,41], which can explain the different changes in their methylation status, in particular, in tumors. In addition, different epigenetic fates of two repeat types in the same tumor can be predetermined by their chromatin structure in normal cells: Sat2 DNA sequences reside in the centromere-adjacent heterochromatin, while 3.3-kb repeats within the D4Z4 array exhibit unexpressed euchromatin-like features [2,42].

Hypomethylation of Sat2 was revealed in tumors with and without hypermethylation of 3.3-kb repeats. The absence of associations between changes of methylation status of two types of DNA repeats confirms the independent role of DNA hypo- and hypermethylation in tumor formation or progression.

### Down-regulation of transcription of DUX-related genes in tumors

Transcriptional repression of *DUX *genes occurred in 68% of cervical carcinomas by methylation and some other mechanism. Proof of transcription of *DUX *genes in some normal cells has been provided [14,17]. We demonstrated that most normal cervical tissues showed clearly detectable, albeit low, levels of transcription from 3.3-kb repeat ORFs. Sequence analysis has demonstrated high homology of the transcripts amplified using our primers to *DUX4, DUX4c*, and *DUX10 *genes (89–100%), but not all transcripts have perfect ORFs. Our findings are consistent with previous studies that indicate that, in spite of hundreds of 3.3-kb copies in the human genome, there are not so many transcripts that could be translated into functional DUX proteins [9,15,17]. Despite many investigations, it is still unclear whether DUX proteins are expressed in normal tissues. Currently, the evidence for the presence of a protein *in vivo *has only been obtained for *DUX4 *and *DUX1 *genes. Both proteins are expressed in human-derived rhabdomyosarcoma cell lines, and DUX4 is expressed in myoblasts of FSHD patients [14-16]. No evidence for the presence of DUX proteins in normal cells has yet been obtained. Surprisingly, the *DUX4 *ORF was recently shown to be conserved over 100 million years of evolution [43]. The authors believe that the *DUX4 *ORF conservation was not occasional but was selected, most likely, for the protein-coding function. High frequency of *DUX4 *transcriptional down-regulation in cervical tumors is an additional indirect evidence for the putative protein-coding function of 3.3-kb repeats and an argument for further search for DUX proteins in normal somatic and embryonic tissues.

### A possible role of hypermethylation and transcriptional silencing of 3.3-kb repeats in cervical cancer

What is the role of hypermethylation and transcriptional silencing of the 3.3-kb repeats in carcinogenesis considering low quantities of functional DUX proteins in normal cells? Some biological functions of DUX proteins are now known. The direct transcriptional target of the DUX4 protein has been identified. DUX 4 interacts *in vitro *with a *cis*-element in the promoter region of paired-like homeodomain transcription factor 1 (*PITX1*) and activates expression of the endogenous *PITX1 *gene in myoblasts [16]. In turn, PITX1 is a positive regulator of p53 and negative regulator of the RAS pathway [44,45]. In addition, DUX4 is a proapoptotic protein, and its forced exogenous expression in cell culture leads to cell death via apoptosis [15]. It is likely that DUX4 might be an initial trigger of a cascade of transcription factors regulating protooncogenes, tumor suppressors, and proapoptotic genes. Low expression of an initial transcription factor frequently occurs in embryonic differentiation. Thus, extremely low concentration of DUX4 in normal cells is not surprising taking into consideration its biological functions, and *DUX4 *silencing in tumors might deregulate several pathways usually affected in them.

An alternative view is that 3.3-kb repeats have a non-coding, regulatory function and play a role in the formation and maintenance of heterochromatin in the subtelomeric regions of chromosomes 4 and 10 or in the maintenance of higher-order chromatin structure and regulation of neighboring gene expression on the chromosomes in normal cells [38,39,46-48]. It was shown that the structure of 3.3-kb repeat chromatin in both 4q and 10q is similar to that of unexpressed euchromatin rather than of constitutive heterochromatin [25,42]. It remains to be established whether the overall increase in the 3.3-kb repeat methylation level in tumors can have an influence on the heterochromatinization of 3.3-kb repeats and/or changes of higher-order chromatin structure and expression of neighboring genes.

## Conclusion

Our results demonstrate that hypermethylation rather than hypomethylation of 3.3-kb repeats is a frequent event in HPV-associated cervical cancer and show a more complex pattern of changes in genome methylation in cancer than previously thought.

## Competing interests

The authors declare that they have no competing interests.

## Authors' contributions

AK carried out the analysis of repeat methylation status, statistical analysis, and contributed to the draft of the manuscript. LP carried out the RNA and DNA extraction and RT-PCR analysis. FL conceived the study and critically revised the manuscript. NK contributed to study concept and design and prepared the draft of the manuscript. All authors have read and approved the final version of the manuscript.

## Pre-publication history

The pre-publication history for this paper can be accessed here:



## Supplementary Material

Additional file 1 **Supplementary Table. Hypermethylation of 3.3-kb repeats and hypomethylation of Sat2 repeats in cervical tumors**. Correlation between hypermethylation of 3.3-kb repeats and hypomethylation of satellite repeats Sat2 are shown in tumor samples. Click here for file
