# Supplementary material for: Hypermethylation of genomic 3.3-kb repeats is frequent event in HPV-positive cervical cancer
Source: BMC Med Genomics. 2009 May 27;2:30. doi: 10.1186/1755-8794-2-30 (PMC2695481; doi:10.1186/1755-8794-2-30)
Supplement: Additional file 1 — Supplementary Table. Hypermethylation of 3.3-kb repeats and hypomethylation of Sat2 repeats in cervical tumors. Correlation between hypermethylation of 3.3-kb repeats and hypomethylation of satellite repeats Sat2 are shown in tumor samples. [file 1755-8794-2-30-S1.pdf]

Supplementary table

Hypermethylation of 3.3-kb repeats and hypomethylation of Sat2 repeats in cervical tumors

№	sample	3.3-kb repeats	Sat2 repeats
1	8	hyper	hypo
2	12	hyper	hypo
3	13	hyper	hypo
4	14	hyper	hypo
5	16	hyper	hypo
6	19		
7	21		hypo
8	22		hypo
9	26		hypo
10	28		hypo
11	30		
12	31		
13	33		hypo
Frequency of changes		5/13	10/13

Numbers of samples are indicated as in Figure 3.
Hyper – hypermethylation, hypo – hypomethylation,
empty box – the absence of changes.
